# High-quality restoration image encryption using DCT frequency-domain compression coding and chaos

**DOI:** 10.1038/s41598-022-20145-3

**Published:** 2022-10-03

**Authors:** Heping Wen, Linchao Ma, Linhao Liu, Yiming Huang, Zefeng Chen, Rui Li, Zhen Liu, Wenxing Lin, Jiahao Wu, Yunqi Li, Chongfu Zhang

**Affiliations:** 1 Zhongshan Institute, School of electronic information, University of Electronic Science and Technology of China, Zhongshan, 528402 China; 2 School of Information and Communication Engineering, University of Electronic Science and Technology of China, Chengdu, 611731 China; 3 Guangdong Provincial Key Laboratory of Information Security Technology, Sun Yat-sen University, Guangzhou, 510006 China

**Keywords:** Computer science, Information technology

## Abstract

With the arrival of the age of big data, the amount and types of data in the process of information transmission have increased significantly, and the full-disk encryption mode used by traditional encryption algorithms has certain limitations of the times. In order to further improve the bandwidth efficiency of digital images in the transmission process and the information effectiveness of digital image transmission, this paper proposes an algorithm of high-quality restoration image encryption using DCT frequency-domain compression coding and chaos. Firstly, the image hash value is used for the generation of an encryption key with plaintext correlation, then lightweight chaos is generated based on the key to obtain a pseudo-random sequence. Secondly, the image is partitioned into subblock, and converted from time domain into frequency domain by employing Discrete Cosine Transform (DCT) on each block, then perform quantization operation based on frequency domain information to obtain DCT coefficient matrix. Thirdly, the direct current (DC) coefficients and alternating current (AC) coefficients are extracted in the DCT coefficient matrix and compressed by different encoding methods to obtain two sets of bitstream containing DC coefficient and AC coefficient information. Fourthly, permute the DC coefficient bit stream by the chaotic sequence, and reconstruct it with the AC coefficient bit stream to obtain the frequency domain ciphertext image. Finally, the chaotic sequence is used to diffuse ciphertext, and the processed hash value is hidden in the ciphertext to obtain the final ciphertext. The theoretical and experimental analysis showed that the key length reaches 341 bits, and the PSNR value of the restored image is close to 60, all of which satisfy the theoretical value. Therefore, the algorithm has the characteristics of high compression rate, high-quality image restoration large key space, strong plaintext sensitivity, strong key sensitivity and so on. Our method proposed in this paper is expected to provide a new idea for confidential and secure communication in the age of big data.

## Introduction

With the continuous development of information technology, the age of big data has arrived^1–6^. While people are enjoying the information dividends, potential information security problems are also gradually exposed. As one of the important media of information transmission in the era of big data, digital image has attracted widespread attention for its security during transmission^7,8^, so it is important to conduct encryption transmission for digital image. However, compared with text information, digital image information has the characteristics of high information redundancy, strong pixel correlation and discrete distribution of key information, etc^9–11^. Most text information security enhancement methods are not appropriate for digital image. Therefore, it is very necessary to study the digital image encryption algorithm. In addition, the amount and type of data are growing rapidly in the age of big data, and the information effectiveness and system throughput in the process of information transmission should also be of concern^12–16^. Therefore, the research on image encryption algorithm under the background of big data era has certain theoretical value and practical significance^17,18^.

Throughout the international research status, digital image encryption has become a hot research topic^19–22^. In recent years, many scholars have devoted themselves to research in this field and achieved good results. In 2018, Ref.^23^ introduce a novel chaos-based image encryption algorithm for color images based on three-dimensional bit-plane permutation. Simulation results and security analyses demonstrate that the algorithm not only has a good encryption effect but can also resist common attacks. In 2019, Ref.^24^ proposed a new algorithm of chaos optical image encryption based on fractional Fourier transform and DNA sequence operation. The experimental results and security analysis show that the algorithm has a good encryption effect and can resist most known attacks. In 2020, Ref.^25^ proposed a new type of time-delay chaotic system, which has different dynamical behavior over time delay. Then based on the system design a novel approach to achieve image encryption. Final experiments illuminate that the given image encryption method has good effectiveness and higher security. In 2021, Ref.^26^ by studying the difference between chaotic sequences and wavelet transform values, proposed a novel technique for digital image encryption and improved previous algorithms. Comparing various performance indexes, it shows that this technique is a suitable choice for actual image encryption. The research results of digital image encryption are far more than these, and a growing number of encryption algorithms are being proposed. From these achievements, we can see that the majority of encryption algorithms have achieved satisfactory results in some aspects, which also greatly promoted the development of information security technology. Unfortunately, in the era of big data, most of the research results have era limitations.

In order to solve the security problem of digital image transmission in the era of big data, some scholars have made continuous contributions to image encryption and compression. For example, Ref.^27–30^ use a variety of different technologies to achieve. The experimental results show that the scheme has more advantages compared to the technology at that time. There are also some scholars who have made pioneering attempts in this direction. For example, in 2020, Ref.^31^ proposed a new multi-image encryption scheme based on quaternion discrete fractional Hartley transform (QDFrHT) and pixel adaptive diffusion. The original images are compressed into four fusion images by Discrete Cosine Transform (DCT) and Zig-Zag operations and then the resulting four images are represented as quaternion algebra. Afterwards, the quaternion signal is processed with the proposed QDFrHT and the double random phase encoding technique. The experimental results show that the scheme is feasible and safe. Unfortunately, most of these studies have the following problems: (1) The use of comprehensive encryption, iterative encryption, multi round encryption and other methods can effectively improve the encryption quality, but there are some problems such as low encryption efficiency and high information redundancy, which have limitations under the background of big data era. (2) The quality of image restoration is often an important indicator to measure the encryption performance, and most of the research focuse on the comparison of plaintext and ciphertext, and lacks the systematic analysis of restored images.

To solve the above problems, this paper proposes an algorithm of high-quality restoration image encryption using DCT frequency-domain compression coding and chaos. Firstly, the encryption key with plaintext correlation is generated by using the image hash value, and the lightweight chaos is generated based on the key to obtain the chaotic pseudo-random sequence. Secondly, divide the image into sub-blocks, and perform DCT on all sub-blocks separately, the image is mapped from time domain to frequency domain, and the quantization operation is carried out based on frequency domain information to obtain DCT coefficient matrix. Then, the direct current(DC) coefficients and alternating current(AC) coefficients in the DCT coefficient matrix are extracted and compressed by different coding methods to obtain two groups of bit streams containing DC coefficients and AC coefficients. Then, the chaotic sequence is used to scramble the DC coefficient bit stream, and the data is reconstructed with the AC coefficient bit stream to obtain the frequency domain ciphertext image. Finally, the ciphertext is diffused using the chaotic sequence, and the processed hash value is hidden in the ciphertext to obtain the final ciphertext. Theoretical and experimental analysis shows that the algorithm has the characteristics of high compression rate, high-quality image restoration large key space, strong plaintext sensitivity, and strong key sensitivity, etc. Therefore, the method proposed in this paper can better improve the effectiveness and reliability of information in the transmission process, and is expected to provide a new idea for secure communication in the context of big data era.

In this paper, we use DCT coding, which requires less arithmetic power, and modify and improve the algorithm with reference to JPEG compression, which has improved the performance compared with traditional JPEG compression. In terms of information encryption, we choose the lightweight chaos with high generation efficiency and low arithmetic power consumption, and introduce the dynamic key associated with plaintext to realize the dynamic encryption process of “one encryption at a time”. In addition, the algorithm is designed based on frequency domain, which can realize selective encryption of information and effectively improve efficiency.

## Correlation theory

### DCT

DCT is a kind of orthogonal transformation^32^. Compared with fast Fourier transform (FFT) and Discrete wavelet transform (DWT), DCT can save arithmetic power and maintain good performance^7^. Let $\{X_{m}|{m=0,1,\ldots ,N-1}\}$ be a signal sequence with length *N*, and 1D discrete chord transform (1D-DCT) is defined as:

1 $$\begin{aligned} Y(u)=C(u)\sqrt{\frac{2}{N}}\sum _{m=0}^{N-1}X(m){\mathrm{cos}} \frac{(2m+1)u\pi }{2N},u=1,2,\ldots ,N-1 \end{aligned}$$

where

2 $$\begin{aligned} C(u)={\left\{ \begin{array}{ll} \frac{1}{\sqrt{2}},u=0\\ 1,u\ne {0}\\ \end{array}\right. } \end{aligned}$$

1D inverse discrete chord transform ( 1D-IDCT ) is defined as:

3 $$\begin{aligned} X(m)=\sqrt{\frac{2}{N}}\sum _{v=0}^{N-1}C(u)Y(u){\mathrm{cos}}\frac{(2m+1)u\pi }{2N} \end{aligned}$$

Extending 1D-DCT to 2D discrete chord transform (2D-DCT)^33,34^. Let $\{X(m,n)|{m=0,1,\ldots ,M-1;n=0,1,\ldots ,N-1}\}$ be two-dimensional signal sequence of $M \times N$, 2D-DCT is defined as:

4 $$\begin{aligned} Y(u,v)=\frac{2}{\sqrt{M \times N}}C(u)C(v)\sum _{m=0}^{M-1} \sum _{n=0}^{N-1}X(m,n){\mathrm{cos}}\frac{(2m+1)u\pi }{2M}{\mathrm{cos}}\frac{(2n+1)v\pi }{2N} \end{aligned}$$

where $$u=1,2,\ldots ,M-1;v=1,2,\ldots ,N-1$$, and

5 $$\begin{aligned}&C(u)={\left\{ \begin{array}{ll} \frac{1}{\sqrt{2}},u=0\\ 1,u\ne {0}\\ \end{array}\right. } \end{aligned}$$

6 $$\begin{aligned}&C(v)= {\left\{ \begin{array}{ll} \frac{1}{\sqrt{2}},v=0\\ 1,v\ne {0}\\ \end{array}\right. } \end{aligned}$$

2D-IDCT is defined as:

7 $$\begin{aligned} X(m,n)=\frac{2}{\sqrt{M \times N}}\sum _{u=0}^{M-1} \sum _{v=0}^{N-1}C(u)C(v)Y(u,v){\mathrm{cos}}\frac{(2m+1)u\pi }{2M}{\mathrm{cos}} \frac{(2n+1)v\pi }{2N} \end{aligned}$$

**Figure 1:**
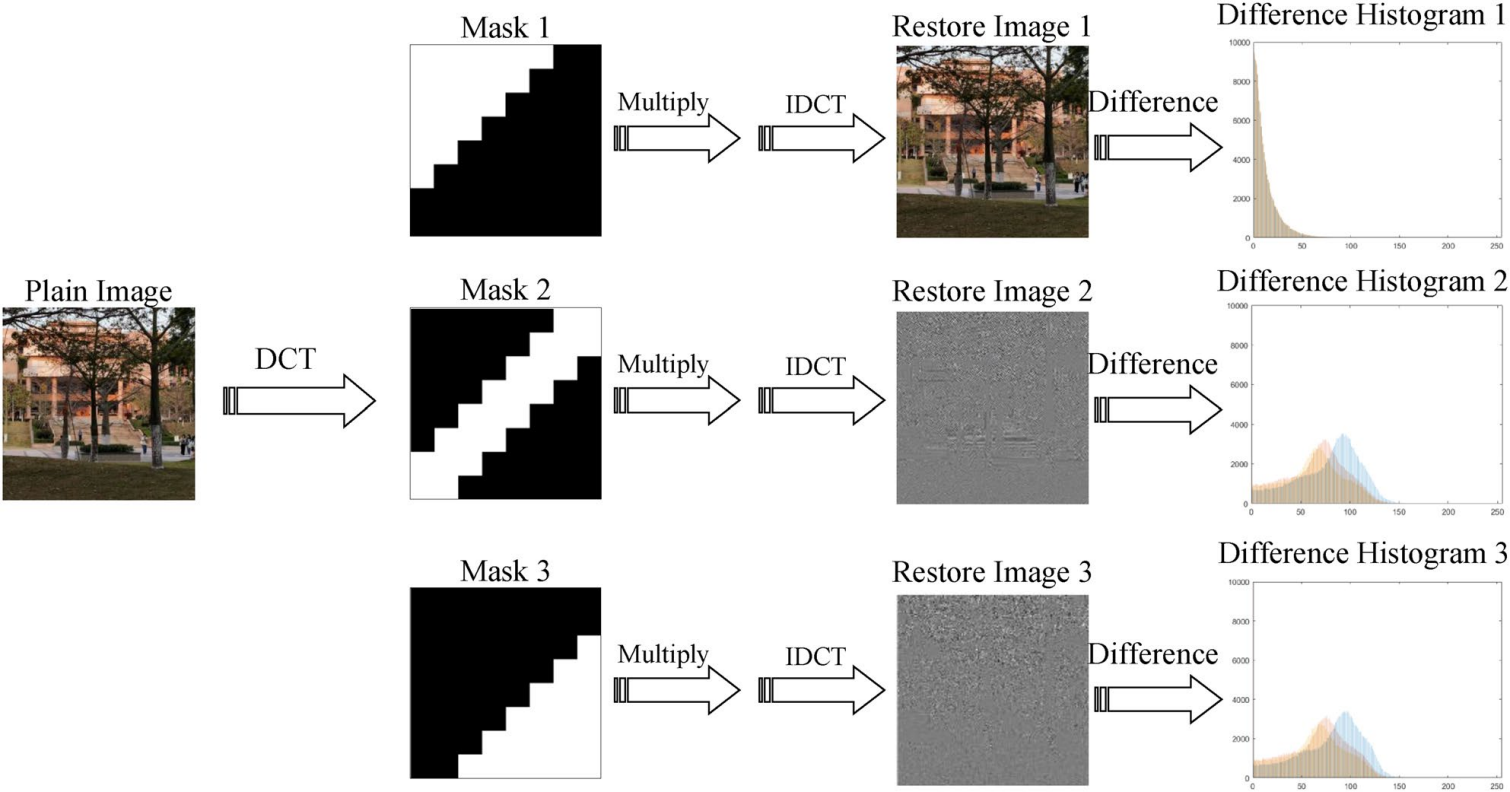
Differential comparison of DCT restoration results under different masks.

Compare with Fourier Transform, DCT in real domain, so digital image processing based on DCT will be more intuitive^35^. In addition, DCT has entropy retention, energy retention, decorrelation, and energy concentration, among which energy concentration is of great significance to digital image encryption^36^. As shown in Fig. 1, the effective information mask with same area and different shape is used to multiply the 2D-DCT image, and the difference analysis is carried out on the image after 2D-DCT. The information retained by the three effective information masks is as follows: the upper left region information, the middle band region information, and the lower right region information. It can be seen from the results of the differential analysis that only Mask 1 can better restore image information, which shows that the energy of the image after DCT is mainly concentrated in the upper left region.

**Figure 2:**
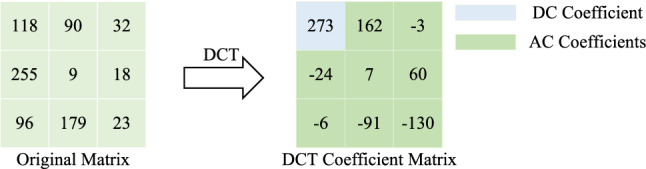
Data comparison before and after DCT.

Further analyze the energy concentration of DCT. DCT is performed on the selected matrix. The data before and after transformation are shown in Fig. 2. The matrix on the right in the figure is the result of rounding after transformation. For the matrix after DCT, it is usually called DCT Coefficient Matrix, and an element in the upper left corner is called DC coefficient, and its remaining elements are AC coefficients. For the DCT coefficient matrix in Fig. 2, the number 273 is the DC coefficient of the coefficient matrix, and it can be seen that the energy of the image after DCT is mainly concentrated in the DC coefficient^2,36^. Based on this, in the digital image encryption, focus on encrypting the DC coefficients in the frequency domain image after DCT, and the ideal encryption effect can be obtained.

In addition, when using DCT to encode the image, the mode of dividing the image into blocks and encoding the sub-blocks and then splicing the sub-blocks is usually adopted. The sub-block size has many choices, and the optimal solution of block size can be obtained from the base image of DCT. For ease of expression, rewrite Eq. (7) as:^37^

8 $$\begin{aligned} X(m,n)=\sum _{u=0}^{M-1}\sum _{v=0}^{N-1}Y(u,v)q(m,n,u,v) \end{aligned}$$

where $m=0,1,\ldots ,M-1;n=0,1,\ldots ,N-1$. It can be seen from the above formula that *X*(*m*, *n*) is composed of ${M\times {}N}$ frequency components, and any frequency component has a specific (*u*, *v*) corresponding to. For each specific (*u*, *v*) value, exhaustive (*m*, *n*) all cases, we will get a matrix, which is the base image of DCT, its mathematical expression is as follows:

9 $$\begin{aligned} X(m,n)=\left[ \begin{matrix} q(0,0,u,v) &{} q(0,1,u,v) &{} \cdots &{} q(0,N-1,u,v)\\ q(1,0,u,v) &{} q(1,1,u,v) &{} \cdots &{} q(1,N-1,u,v)\\ \vdots &{} \vdots &{} \vdots &{} \vdots \\ q(M-1,0,u,v) &{} q(M-1,1,u,v) &{} \cdots &{} q(M-1,N-1,u,v)\\ \end{matrix}\right] \end{aligned}$$

**Figure 3:**
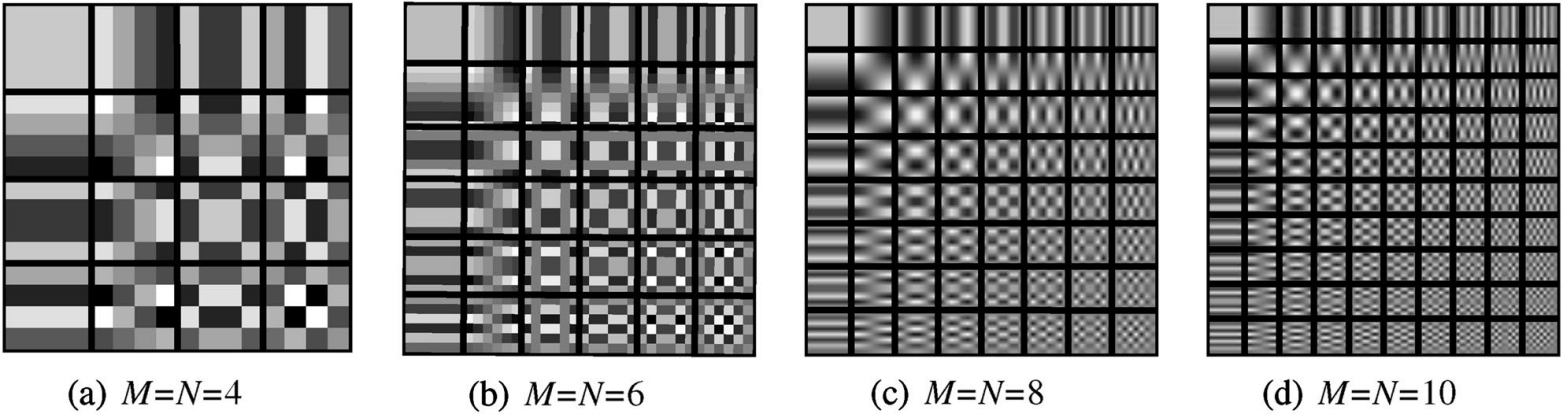
DCT base images.

The base images corresponding to different (*u*, *v*) values have ${M\times {}N}$ amplitudes, and they are independent of *X*(*m*, *n*). DCT base images with different *M* and *N* values are shown in Fig. 3. The base image can reflect main features of the transformation, as can be seen when ${M=N \ge 8}$, the performance of DCT base image meets the expectations^38^. Therefore, the $8 \times 8$ block mode can reduce the computational complexity of DCT to the greatest extent under the condition of ensuring accuracy, which is the optimal solution for block size selection^39^.

### Chaotic system

The chaotic system was first proposed by American meteorologist Lorenz in 1963^40^, which is a nonlinear system with non-divergence, non-convergence, and non-periodic characteristics. Due to the complex dynamic principle of chaotic system, the sequences generated by the system usually have strong randomness. At the same time, because the chaotic system is highly sensitive to the initial value, the sequence is usually difficult to predict, so the chaotic system is widely used in secure communication.

Lorenz chaotic system, as the first continuous chaotic system, has the advantages of simple form and high generation efficiency. However, the cryptosystem using Lorenz chaotic sequence as the key usually has the problems of small key space and poor anti-attack ability. Therefore, a general framework of 1-D chaotic maps called the Dynamic Parameter-Control Chaotic System(DPCCS) was proposed by Ref.^41^. DPCCS is able to produce a huge number of new chaotic maps. Evaluations and comparisons show that chaotic maps generated by DPCCS are very sensitive to their initial states, and have wider chaotic ranges, better unpredictability and more complex chaotic behaviors than their seed maps.

**Figure 4:**
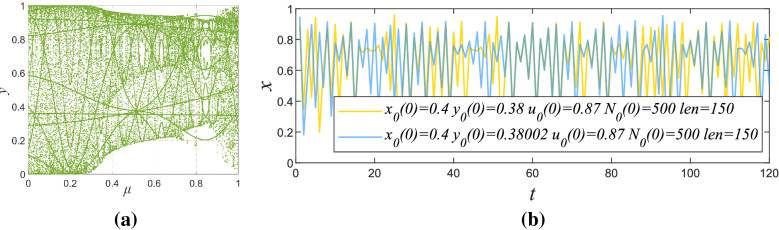
(**a**) Bifurcation diagrams of the SCL maps; (**b**) Sequence comparison before and after $y_{0}(0)$ perturbation.

This paper uses a sine map to control the parameter of the logistics chaotic map, the chaotic system is simply called Sine-control-Logistic(SCL). Since SCL chaos is a lightweight discrete chaotic system, it does not require sampling and other operations in use, which is more convenient than the continuous chaotic system. SCL chaotic system is defined as:

10 $$\begin{aligned} x_{n+1}=4(1-0.1y_{n+1})x_{n}(1-x_{n}) \end{aligned}$$

where

11 $$\begin{aligned} y_{n+1}=\mu {{\mathrm{sin}}(\pi {y_{n}})} \end{aligned}$$

where *n* and ${n}+1$ are used as cell markers, $x_{n}$ and $y_{n}$ are system initial values, $\mu$ is the control values of $y_{n+1}$, $y_{n+1}$ is the control values of $x_{n+1}$, $x_{n+1}$ is the sequence generated by chaos. When $u\in (0.87,1)$, $x_{n}\in (0.35,0.45)$, $y_{n}\in (0.35,0.45)$ the system is in a chaotic state. Figure 4 is the SCL maps and the initial value sensitivity of SCL system.

### Compressed encoding

**Figure 5:**
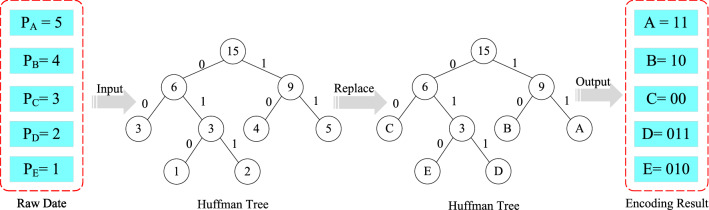
Huffman coding process.

Digital image usually has the characteristics of a large amount of data and high information redundancy, which leads to low spectral efficiency and limited information validity in the transmission process. Therefore, it is necessary to find a method to remove image information redundancy and improve the effectiveness of transmission information. In this paper, compression coding is used to achieve this goal. There are three coding methods, namely:

Huffman Coding: Huffman Coding is a lossless compression coding^42^. The encoding steps can be summarized as follows: Firstly, the probability of image pixel values is arranged in descending order. Secondly, the pixel values are added sequentially in order until the final sum of probabilities is 1. Finally, the path of each pixel value is drawn from the probability of 1, and 0 and 1 are recorded in the order of paths. The final binary code is the Huffman code of pixels. Set A, B, C, D, E five characters, the frequency of occurrence is 5, 4, 3, 2, 1 respectively. Then the coding results are shown in Fig. 5, where P represents the frequency of the characters.

**Figure 6:**
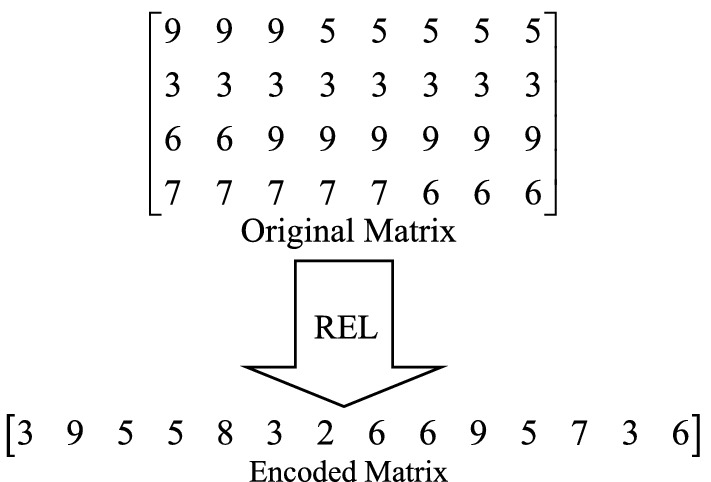
RLE process.

Run-Length Encoding (RLE): RLE is a lossless coding method and is commonly used in digital image compression^43^. The encoding principle is that the adjacent pixels with the same pixel value in a row are represented by two bytes. As shown in Fig. 6, the first byte records the number of repetitions of pixels, and the second byte records the specific pixel value. The effect of RLE mainly depends on the characteristics of the image itself: the larger the pixel block of the same pixel in the image, the better the compression effect and the higher the compression ratio. On the contrary, the compression effect is poor.

Differential Pulse Code Modulation (DPCM): DPCM is a kind of linear predictive coding and a lossy compression coding^44^. The main principle is: using the past sampling values to predict the current sampling values, and coding their difference. For image signals, the instantaneous slope of the signal is so large that it is easy to cause overload, simple increment modulation cannot be used for coding. Therefore, a modulation method that combines the characteristics of incremental modulation and pulse code modulation is usually used for coding, which is called DPCM. Assuming that the discrete-time analog signal is set ${X_{k}}$, the signal value at time *K* is $X_{k}$, and the linear combination of the past *N* signals is used to predict, then the predicted value $\hat{X_{k}}$ is:

12 $$\begin{aligned} \{\hat{X_{k}}\}=\sum _{i=1}^{N}a_{i}X_{K-1} \end{aligned}$$

There is an information difference $e_{k}$ between the actual value $X_{k}$ and the predicted value $\hat{X_{k}}$, that is:

13 $$\begin{aligned} e_{k}=X_{k}-\hat{X_{k}}=X_{k}-\sum _{i=1}^{N}a_{i}\hat{X_{K-1}} \end{aligned}$$

If appropriate *N* and $a_{i}$ are selected to make the characteristic of $e_{k}$ a white noise process with an average value of 0 and recorded as $W_{K}$, then the restored $X_{K}$ is:

14 $$\begin{aligned} X_{K}=\sum _{i=1}^{N}a_{i}\hat{X_{K-1}}+W_{K} \end{aligned}$$

## The proposed encryption algorithm

**Figure 7:**
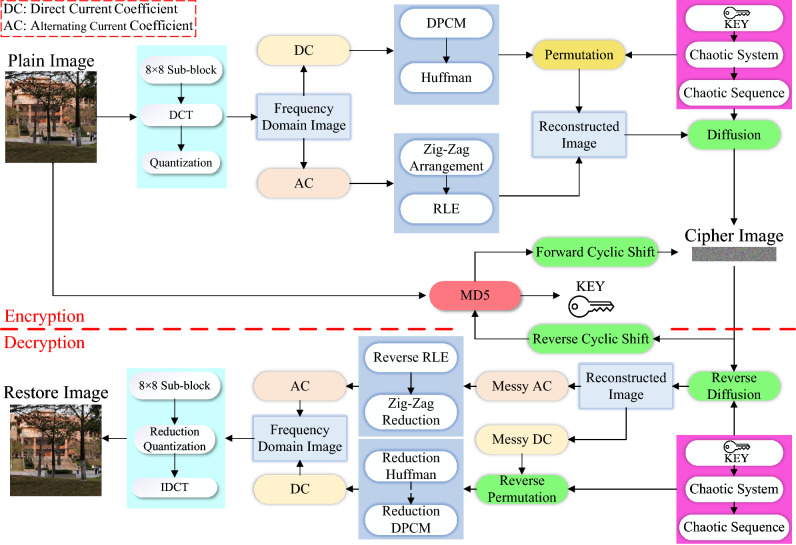
Principle and mechanism of image encryption.

The current chaotic encryption algorithms mostly use the static key encryption mode for complete images. Such methods usually have problems such as poor security performance and low effectiveness of information transmission, and have limitations under the background of big data era. Therefore, this paper proposes a frequency domain compression encryption algorithm based on lightweight chaos, and introduces a dynamic key with plaintext correlation. The specific process of encryption and decryption is shown in Fig. 7. This algorithm uses the dynamic key to realize the dynamic encryption mode of “one image and one encryption”, and the dynamic key is hidden during the transmission process. The specific encryption steps are as follows:

$\mathbf{Step 1}$: Dynamic Key Generation and Chaotic Sequence Generation

Read in the plaintext and use the hash table to obtain the MD5 hash value of the plaintext image, and encode the 16-bit hash value into 4 decimal numbers that conform to the initial chaotic value interval. The specific encoding rules are as follows:

15 $$\begin{aligned} {\left\{ \begin{array}{ll} x_{1}(0)=0.35+(m_{1}\oplus {}m_{2}\oplus {}m_{3}\oplus {}m_{4})/2560\\ y_{1}(0)=0.35+(m_{5}\oplus {}m_{6}\oplus {}m_{7}\oplus {}m_{8})/2560\\ x_{2}(0)=0.35+(m_{9}\oplus {}m_{10}\oplus {}m_{11}\oplus {}m_{12})/2560\\ y_{2}(0)=0.35+(m_{13}\oplus {}m_{14}\oplus {}m_{15}\oplus {}m_{16})/2560\\ \end{array}\right. } \end{aligned}$$

where $\oplus$ is bitwise XOR operation, $m_{1-16}$ is the result of bitwise read for MD5 hash, $x_{1}(0),y_{1}(0),x_{2}(0),y_{2}(0)$ is the initial value of chaotic system. Generating a chaotic system by using the initial chaotic value can receive two chaotic sequences: $K_{1},K_{2}$ for encryption. In addition, in order to ensure the security of the hash value, a bit-level cyclic shift is performed on the hash value, and the result after cyclic shift is re-encoded into decimal number every 8 bits. The specific formula is as follows:

16 $$\begin{aligned} {\left\{ \begin{array}{ll} hash2=\mathrm{circshift}({{ hash}},[0,-7])\\ m=\mathrm{blkproc}({{ hash}}2,[1,8],\mathrm{'two2ten'})\\ \end{array}\right. } \end{aligned}$$

where circshift$(\cdot )$ is the function for circular shift, blkproc$(\cdot )$ is the function for segmentation, *hash* is binary hash value and its length is 128bit, matrix [0, − 7] indicates that the cyclic shift operation is unchanged for row elements, rotate column elements 7 units to the left, *hash*2 is the circularly shifted binary hash value, $\mathrm{'two2ten'}$ is the customized function for shift binary to decimal, matrix [1,8] indicated encode once per 8 elements, *m* is the decimal number after hash value been bit cyclic shifted.

$\mathbf{Step 2}$: Time Domain Shifting and DCT Coding

**Figure 8:**
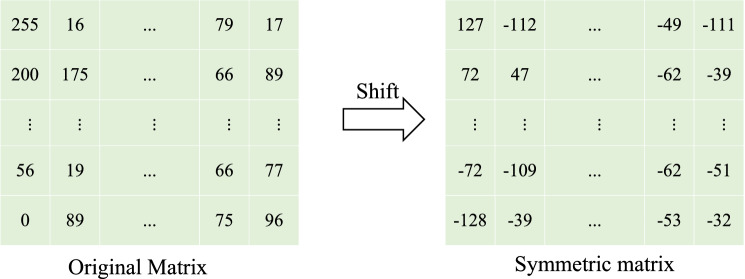
Pixel value translation.

Since DCT requires that the definition domain of the function is symmetrical, as shown in Fig. 8, since DCT requires that the definition domain of the function is symmetrical, the image pixel values are shifted right in the time domain, the shifted pixel values are distributed between − 128 and 127. Subsequently, the image is divided into several $8\times {8}$ sub-blocks, and DCT is performed on these sub-blocks respectively to map the image matrix from the spatial domain to the frequency domain.

$$\mathbf{Step 3}$$: Quantify

Quantify the sub-block from $\mathbf{Step 2}$ respectively. The specific formula is:

17 $$\begin{aligned} I'=\mathrm{round}\left( \frac{{ I}}{{ Q}}\right) \end{aligned}$$

where round($\cdot$) is the rounding function, *I* is a sub-block image of size $8\times {}8$, ${I'}$ is the matrix after quantify, and *Q* is the quantization matrix. The quantization matrix is the key to controlling the compression ratio and also the image recovery after DCT. The quantization matrix can be customized according to the quality requirements of the output image. Usually, the custom quantization matrix is proportional to the standard quantization matrix. The larger the number in the matrix, the lower the image quality and the higher the compression rate. The commonly used standard quantization matrix $Q_{Y}$ is:

18 $$\begin{aligned} Q_{Y}=\left[ \begin{matrix} 16 &{} 11 &{} 10 &{} 16 &{} 24 &{} 40 &{} 51 &{} 61\\ 12 &{} 12 &{} 14 &{} 19 &{} 26 &{} 58 &{} 60 &{} 55\\ 14 &{} 13 &{} 16 &{} 24 &{} 40 &{} 57 &{} 69 &{} 56\\ 14 &{} 17 &{} 22 &{} 29 &{} 51 &{} 87 &{} 80 &{} 62\\ 18 &{} 22 &{} 37 &{} 56 &{} 68 &{} 109 &{} 103 &{} 77\\ 24 &{} 35 &{} 55 &{} 64 &{} 81 &{} 104 &{} 113 &{} 92\\ 49 &{} 64 &{} 78 &{} 87 &{} 103 &{} 121 &{} 120 &{} 101\\ 72 &{} 92 &{} 95 &{} 98 &{} 112 &{} 100 &{} 103 &{} 99\\ \end{matrix}\right] \end{aligned}$$

$\mathbf{Step 4}$: Coefficient Extraction and Compression Coding

Extract the DC coefficients from each sub-block and construct these coefficients as a row matrix. Perform DPCM and Huffman Coding on the row matrix successively, and finally obtain a binary bit stream based on DC coefficients. Extract the AC coefficients from each sub-block, press Zig-Zag operations and perform RLE, and finally get a binary bit stream based on AC coefficients.

$\mathbf{Step 5}$: Coefficient Scrambling and Cipher Text Construction

Use the chaotic sequence ${K_{1}}$ to perform column permutation on the DC coefficient bit stream, the specific formula is as follows:

19 $$\begin{aligned} {\left\{ \begin{array}{ll} {[}1,W]=\mathrm{size}({ DC})\\ {[}sW,indexW]=\mathrm{sort}({ K}_{{ 1}})\\ P(1,i)=DC(1,indexW(i))\\ \end{array}\right. } \end{aligned}$$

where size($\cdot$) is the array size read function, sort($\cdot$) is the sorting function, *W* is the width of the matrix, *DC* is the DC coefficient bit stream obtained in $\mathbf{Step 4}$, *sW* and *indexW* are the sorting results and sorting index of the sort($\cdot$) function, respectively, *P* is the permuted DC coefficient bit stream. The replaced DC coefficient bit stream and the AC coefficient bit stream obtained in $\mathbf{Step 4}$ are numerically reconstructed, and they are constructed into cipher text images with the same length and different width as the plaintext images.

$\mathbf{Step 6}$: Diffusion Encryption and Key Hiding

**Figure 9:**
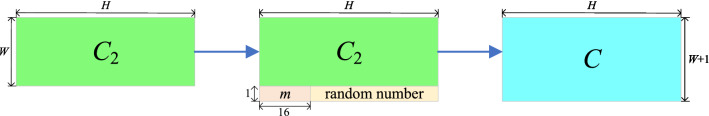
Hash value hiding.

The matrix constructed in $\mathbf{Step 5}$ is diffusion encrypted by using chaotic sequence $K_{2}$, the specific formula is as follows:

20 $$\begin{aligned} {\left\{ \begin{array}{ll} {[}H,W]=\mathrm{size}({ C}_{{ 1}})\\ R=\mathrm{floor}(\mathrm{mod}({{ K}_{{ 2}}}\times 10^{10},256))\\ R_{1}=\mathrm{reshape}({ R},{ H},{ W})\\ C_{2}=\mathrm{bitxor}({ R}_{{ 1}},{ C}_{{ 1}})\\ \end{array}\right. } \end{aligned}$$

where floor($\cdot$) and mod($\cdot$) are the integer-valued function and complementary function, bitxor($\cdot$) is the bit-level XOR function, reshape($\cdot$) is the matrix rearrangement function, $C_{1}$ is the cipher text from $\mathbf{Step6}$, *H* and *W* are the length and width of $C_{1}$, and *R* is the processed chaotic sequence. The method of key hiding is shown in Fig. 9. $C_{2}$ is the ciphertext obtained by Eq. (20), *C* is the final output ciphertext, and *m* is the key value obtained in $\mathbf{Step1}$.

## Experimental verification and discussion

In this paper, the proposed encryption algorithm is verified and analyzed on MATLAB 2019. The system runs on a PC with Windows 10 64-bit operating system, Intel(R) Core(TM) i7-7700HQ CPU @ 2.80GHz 2.80 GHz processor and 16G RAM. In order to ensure the rigor of the experimental verification process, most of the test images in this paper are selected from the “USC-SIPI Image Database”^45^ and “Wallpapers”^46^ as the test images.

### NIST 800-22 Test

Special Publication 800-22 Test Kit (NIST Randomness Test) provided by the National Institute of Standards and Technology^47^. The NIST test program is a statistical package that includes 16 test methods. These tests test the randomness of arbitrarily long binary sequences generated by hardware and software used as a confidential random or pseudo-random number generator. The binary sequence generated by the chaotic system we are using successfully passes this test, and the test results are shown in Table 1.

**Table 1:** NIST-800-22 test results.

Statistical tests	p-values	Results
Frequency (monobit) test	0.191687	Successful
Block-frequency test	0.102526	Successful
Cumulative-sums test	0.162606	Successful
Runs test	0.657933	Successful
Longest-run test	0.637119	Successful
Binary matrix rank test	0.350485	Successful
Discrete fourier transform test	0.739918	Successful
Non-overlapping templates test	0.007694	Successful
Overlapping templates test	0.574903	Successful
Maurer’s universal statistical test	0.964295	Successful
Approximate entropy test	0.834308	Successful
Random-excursions test (x = − 4)	0.000648	Successful
Random-excursions variant test (x = − 9)	0.048716	Successful
Serial test-1	0.637119	Successful
Serial test-2	0.699313	Successful
Linear-complexity test	0.616305	Successful

### Key space

Since the chaotic system is highly sensitive to the initial chaotic values and control parameters, in this paper, four initial chaotic values $x_{1}(0),y_{1}(0),x_{2}(0),y_{2}(0)$ are used as the key parameters for encryption and decryption. The key parameter selects the double-precision data type with the precision of $10^{-16}$, and the key space capacity is $10^{16\times {}4}\approx {}2^{213}$. In addition, this paper introduces the MD5 hash value associated with the original image in this space to optimize the key space. The generated 128-bit hash value can expand the key space to $2^{213+128}=2^{341}$. Finally, the key length reaches 341 bits. The results compared with other literature are shown in Table 2. It can be seen that the algorithm in this paper has a larger key space compared to other algorithms, which is sufficient to resist brute-force attacks^48^.

**Table 2:** Key space comparison.

	Ref.^49^	Ref.^50^	Ref.^51^	This paper
Key space (bit)	256	299	309	341

### Image restoration quality analysis

In the compression algorithm, the information loss of the image is unavoidable, so it is necessary to analyze the quality of the decrypted and restored image. It is worth noting that the quantization matrix *Q* used in this section is an all-1 matrix. The reduction quality analysis in this paper is analyzed from four aspects: Unified Average Changing Intensity (UACI), Mean Square Error (MSE), Peak Signal-to-Noise Ratio (PSNR), and Structural SIMilarity (SSIM). They are as follows:

21 $$\begin{aligned} {\left\{ \begin{array}{ll} \mathrm{UACI}=\frac{1}{{ H}\times {}{} { W}}\sum \nolimits _{{{ i}={ 1}}}^{{{ H}}} \sum \nolimits _{{{ j}={ 1}}}^{{{ W}}}\frac{|{{ I}({ i},j)-C(i,j)}|}{255} \times {}100\%\\ \mathrm{MSE}=\frac{1}{{ H}\times { W}}\sum \nolimits _{{ i}={ 1}}^{{ H}} \sum \nolimits _{{ j}={ 1}}^{{ W}}\big (I(i,j)-C(i,j)\big )^2\\ \mathrm{PSNR}=10\lg \left( \frac{{ MAX}_{{ I}}^{ 2}}{{ MSE}}\right) \\ \mathrm{SSIM}=\frac{[2{\mu _{{ I}}\mu _{{ P}}}+(0.01{{ L}})^2] {[}2{\sigma _{{ IP}}}+(0.03{{ L}})^2]}{[{\mu _{{ I}}}^2 +{\mu _{{ P}}}^2+(0.01{{ L}})^2][{\sigma _{{ I}}}^2 +{\sigma _{{ P}}}^2+(0.03{{ L}})^2]} \end{array}\right. } \end{aligned}$$

where *H* and *W* are the length and width of the image respectively, *I* is the plaintext, *C* is the image after restoration, ${{ MAX}_{{ I}}}$ is the maximum value of the image pixel, and ${{ MAX}_{{ I}}}$ = 255 in the grayscale image.$\mu _{I}$ and $\mu _{P}$ are the average values of the plaintext image and the image after restoration, $\sigma _{I}$ and $\sigma _{P}$ are the variances of the plaintext image and the image after restoration, $\sigma _{IP}$ is the covariance of the plaintext image, and *L* is the dynamic range of pixel values. For two identical images, the theoretical value of UACI and MSE is 0, the theoretical value of SSIM is 1, and the PSNR should approach positive infinity. For digital images, it is generally considered that when the PSNR is 60dB, the image is numerically undistorted, and when the PSNR is 40dB, the image distortion is difficult to detect. In addition, the compression ratio defined in this paper is:

22 $$\begin{aligned} C=\left( 1-\frac{I_{C}}{I_{P}}\right) \times 100\% \end{aligned}$$

where *C* is the compression rate, ${I_{C}}$ is the data of the ciphertext image, and ${I_{P}}$ is the data of the plaintext image. In the encryption algorithm of this paper, since the plaintext and ciphertext are images of the same length and different width, and the length of each pixel is 8 bits, the above formula can be simplified as:

23 $$\begin{aligned} C=\left( 1-\frac{H_{C}}{H_{P}}\right) \times 100\% \end{aligned}$$

where ${H_{C}}$ is the pixel length of the ciphertext image, ${I_{C}}$ is the pixel length of the plaintext image.

The experimental data are shown in Table 3. From the experimental results, it can be seen that the value of UACI is around 0.033, the value of MSE is around 0.084, the value of PSNR is around 58.9, and the value of SSIM is close to 1, all of which satisfy the theoretical value. Therefore, the algorithm in this paper can ensure that after encryption and compression Restore the high quality of the image.

The algorithm in this paper is compared with other algorithms and traditional JPEG and JPEG2000, some experimental results are shown in Table 4. It can be seen that this algorithm has certain advantages over other algorithms when the compression rate is constant.

**Table 3:** Image restoration quality analysis.

Pictures	Size	Compression ratio (%)	UACI (%)	MSE	PSNR (dB)	SSIM
5.1.14.tiff^45^	$256\times {}256$	22.2656	0.0331	0.0844	58.8668	0.9997
7.1.10.tiff^45^	$512\times {}512$	31.4453	0.0328	0.0837	58.9038	0.9995
5.3.10.tiff^45^	$1024\times {}1024$	30.2734	0.0323	0.0836	58.9064	0.9995
river.tiff^46^	$3840\times {}2160$	33.6574	0.0177	0.0452	61.5837	0.9998

**Table 4:** PSNR at constant compression rate: 80%.

	This paper	Ref.^52^	JPEG	JPEG2000
Pepper^45^	38.00	38.93	35.05	35.27
Baboon^45^	37.46	30.69	30.89	28.78
Boat^45^	35.66	38.26	34.52	31.32
Cameraman^45^	45.03	34.82	45.91	28.97

### Comparison of decrypted images under different compression ratios

The quantization matrix is the key to controlling the compression ratio (encryption operations do not affect the compression ratio). The compression rate can be changed by customizing the quantization matrix. The relationship between the custom quantization matrix and the standard quantization matrix is as follows:

24 $$\begin{aligned} Q=\alpha {}Q_{Y} \end{aligned}$$

where *Q* is the custom quantization matrix, ${Q_{Y}}$ is the standard quantization matrix, the specific value of the matrix is given in $\mathbf{Step 3}$ of the algorithm design, $\alpha$ is the quantization scale factor, and the value in this paper is $\alpha =\{2^{-4},2^{-3},2^{-2},2^{-1},1,2\}$. Usually, the larger the $\alpha$, the higher the compression rate, the quality of the restored image is lower. When the PSNR is 40dB, the distortion of the image is difficult to detect, so PSNR$\geqslant$40dB is used as the index of qualified image quality.

**Figure 10:**
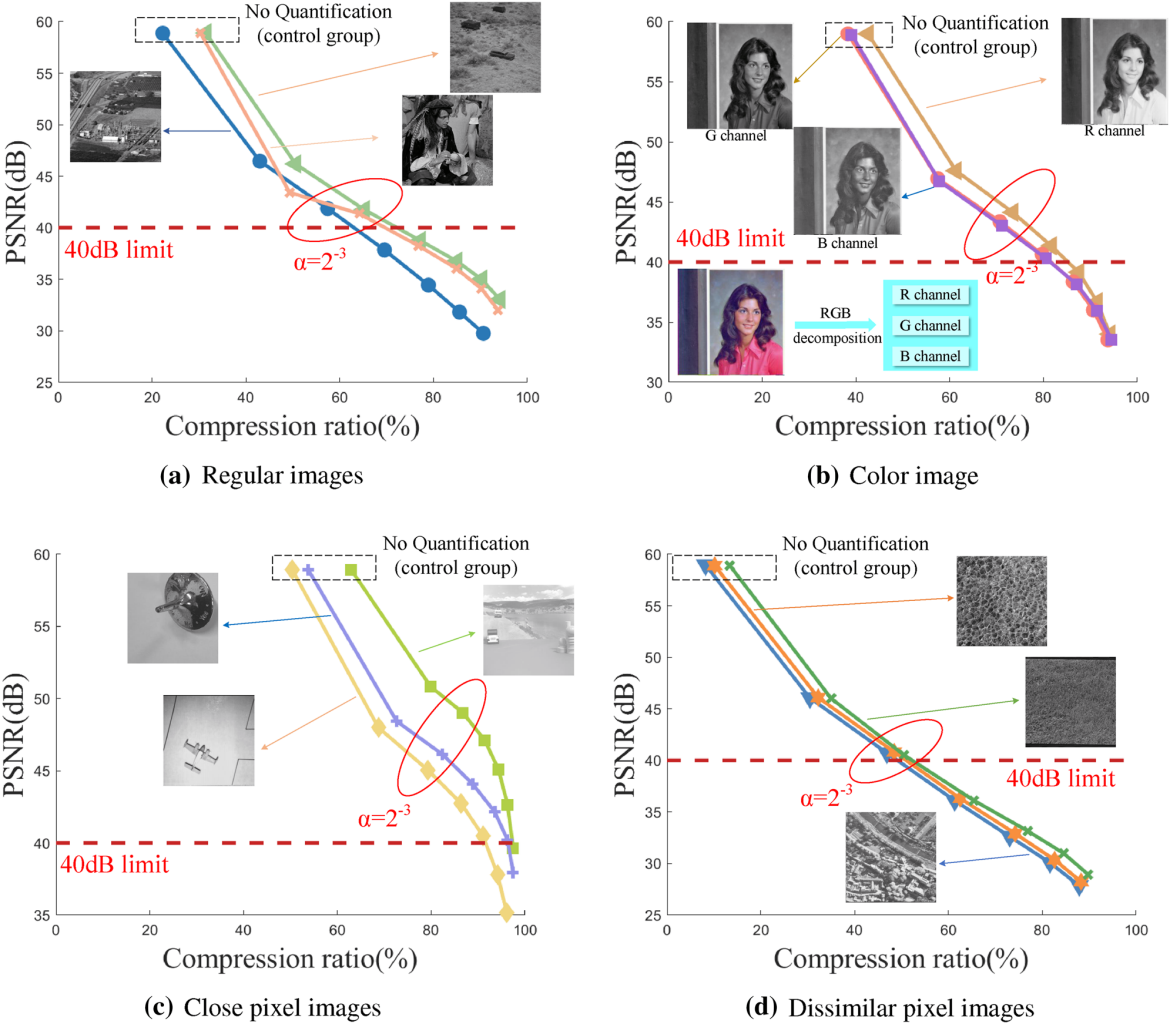
System performance analysis under different $\alpha$.

By adjusting the quantization scale factor $\alpha$, observe the compression of different images, and then evaluate the quality of the decrypted image through PSNR to find the optimal value of the quantization scale factor $\alpha$. The experimental results divide the images into four categories: general images, color images, images with similar pixels, and images with large pixel differences. The experimental results are shown in Fig. 10. The quantization scale factorin $\alpha$ in Fig. 10 is $2,1,2^{-1},2^{-2},2^{-3},2^{-4}$ from left to right. When $\alpha =2^{-3}$, the PSNR value of the decrypted image is about 40dB, the image compression rate is between 50 and 85% at this time, and the compression rate is better. Based on the above, the subsequent experimental analysis in this paper, the value of the quantitative scale factor $\alpha$ is uniformly specified as $2^{-3}$.

### Sensitivity of the original image

**Figure 11:**
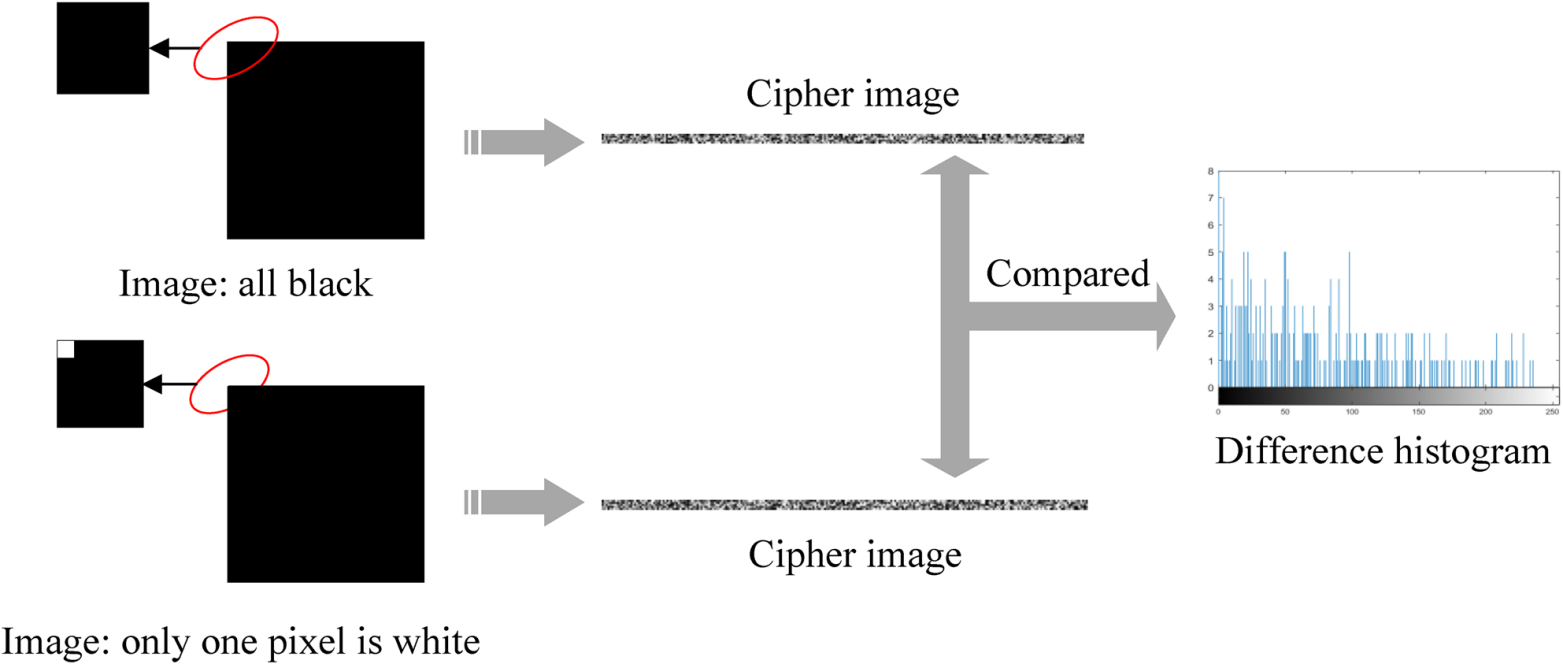
Plaintext sensitivity analysis.

The encryption algorithm in this paper has strong plaintext sensitivity. When there is only a slight difference between two plaintext images, the encrypted ciphertext images will show a huge difference. The experimental results are shown in Fig. 11. The following is a differential analysis of the experimental results: two plaintext images with a size of $255\times {}255$ that differ by only one-pixel value, the encrypted ciphertext images are both $5\times {}255$ in size, with a total of 1275 pixels, there are 261 different pixels in total, that is, there is a 20% difference between the two ciphertexts. Experiments show that the encryption algorithm in this paper has better sensitivity to plaintext, and has better performance in blocking chosen-plaintext attacks.

### Correlation analysis of adjacent pixels

Correlation analysis is a method for judging the merits of digital image encryption algorithms in eliminating pixel correlation in plaintext images^53^. Due to the large amount of information redundancy, digital plaintext images have strong correlations between adjacent pixels in the horizontal, vertical and diagonal directions, which is unfavorable for information security. The digital image encryption algorithm is designed to eliminate the strong correlation between pixels, so the encryption algorithm usually compares the pixel correlation of the plaintext image and the ciphertext image in the horizontal, vertical, and diagonal directions when analyzing the security performance.

**Figure 12:**
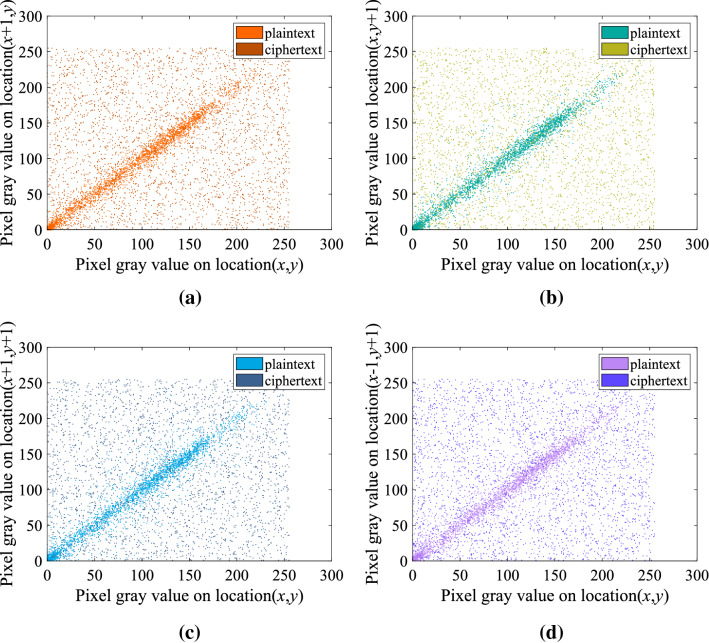
Correlation coefficients distribution map of plain image and cipher image of 5.2.10.tiff (**a**) horizontal correlation; (**b**) vertically correlation; (**c**) diagonal correlation; (**d**) against angular direction correlation.

Taking the image 5.2.10.tiff^45^ as an example, randomly select 3000 pairs of adjacent pixels in the plaintext image and the ciphertext image, and calculate the adjacent pixel correlation coefficient in the horizontal, vertical, diagonal and anti-diagonal directions respectively. The correlation scatters plot in each direction is shown in Fig. 12. From the experimental results, it can be seen that the adjacent pixels in the horizontal, vertical, diagonal, and anti-diagonal directions of the plaintext image are centrally distributed, while in the ciphertext image, the adjacent pixels in these directions are all randomly distributed. The experimental results show that the adjacent pixels of the plaintext image are highly correlated, while the adjacent pixels of the ciphertext image encrypted by this algorithm has almost no correlation, indicating that the encryption algorithm has high security.

### Histogram analysis

Cryptographic images need better statistical properties to resist attacks against encrypted images^54^. Image histogram can effectively represent the information carried by digital images through the gray value distribution of pixels. Statistical decryption attacks often use this as a breakthrough point to crack encrypted images. Digital image encryption algorithm can encrypt the histogram of plaintext image to the histogram of noise style with uniform distribution characteristics to cover up the main information of plaintext digital image. The more uniform the histogram is, the better the main information of plaintext digital image is hidden.

**Figure 13:**
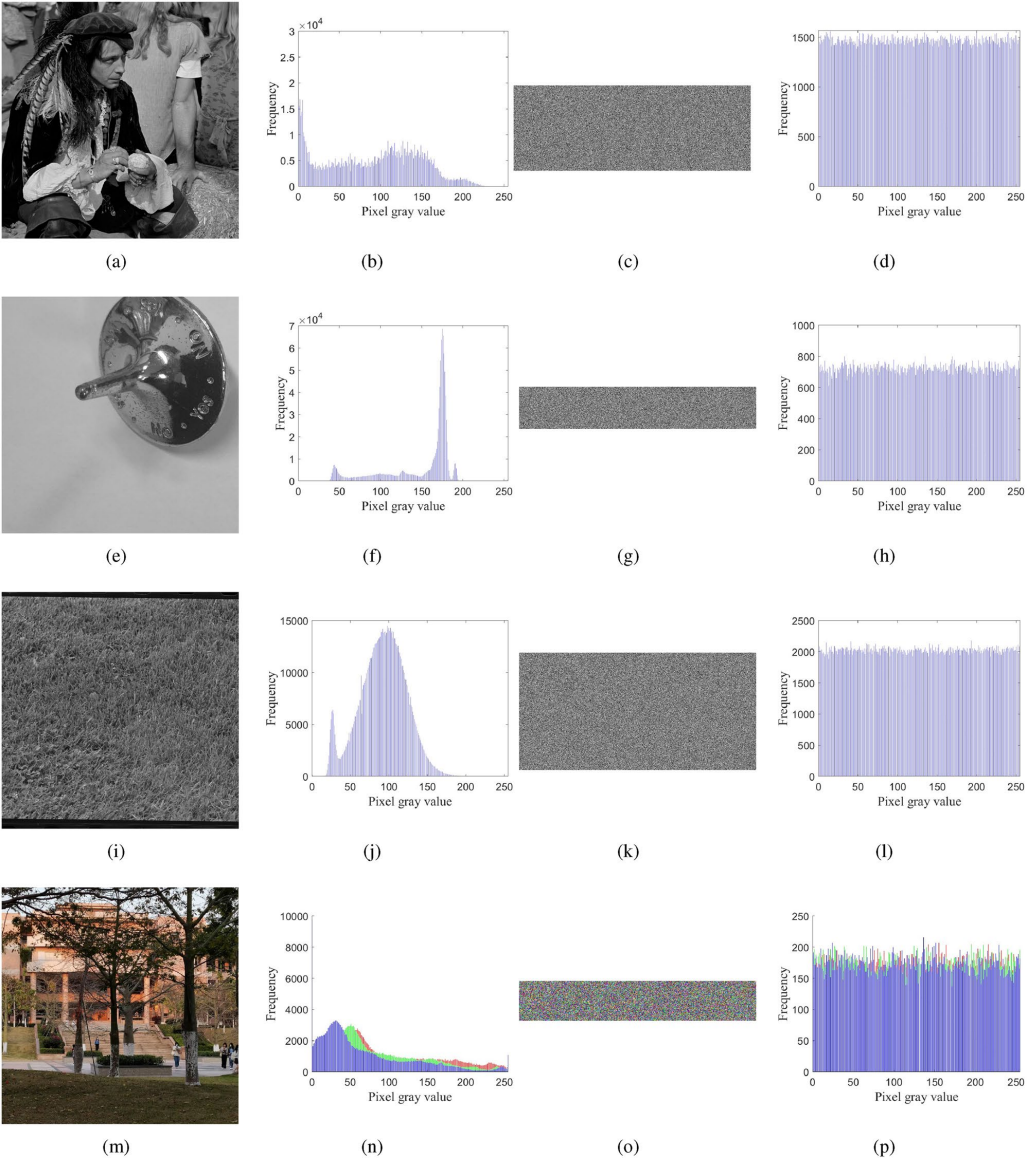
The histograms of images before and after encryption (**a**) $1^{\#}$^45^ plain image; (**b**) the histogram of (**a**); (**c**) $1^{\#}$ cipher image; (**d**) the histogram of (**c**); (**e**) $2^{\#}$ plain image; (**f**) the histogram of (**e**); (**g**) $2^{\#}$ cipher image; (**h**) the histogram of (**g**); (**i**) $3^{\#}$ plain image; (**j**) the histogram of (**i**); (**k**) $3^{\#}$^45^ cipher image; (**l**) the histogram of (**k**); (**m**) $4^{\#}$ plain image; (**n**) the histogram of (**m**); (**o**) $4^{\#}$ cipher image; (**p**) the histogram of (**o**).

Different images are selected as plaintext images for analysis, and the results are shown in Fig. 13. Images $1^{\#}$ and $3^{\#}$ are freely available in Image Library “USC-SIPI Image Database”^45^. Images $2^{\#}$ and $4^{\#}$ are taken by author Linchao Ma. It can be seen from the experimental results that any image after encryption processing is noise-like distribution, which shows that the encryption scheme can better resist statistical attacks and has a better encryption effect.

### Information entropy analysis

Information entropy is an indicator used to measure the information content and uncertainty of digital images^55^. The greater the information entropy of digital image, the higher the information uncertainty of digital image, and the more invisible information contained in digital image. Therefore, in the digital image encryption algorithm, it is usually hoped that the ciphertext image has a large information entropy. The mathematical calculation formula of information entropy is as follows:^56^

25 $$\begin{aligned} H(n)=-\sum _{i=1}^{L}P(n_{i})\log _{2}P(n_{i}) \end{aligned}$$

where *i* represents the pixel gray value, $P(n_{i})$ represents the probability that the gray value appears in the digital image. Through the calculation of Eq. (25), it can be seen that for 8bit gray image, the theoretical value of information entropy is the maximum value of 8. Taking different images as test images, the test results are shown in Table 5. The experimental results show that the information entropy of both ciphertext images is above 7.998, which is close to the theoretical value of 8, indicating that the encryption algorithm has a good encryption effect and can better resist the information entropy attack.

**Table 5:** Information entropy of the plain image and ciphered image.

Picture	Plain image	Cipher image
7.1.10.tiff^45^	5.9088	7.9981
1.1.13.tiff^45^	7.2955	7.9986
5.2.10.tiff^45^	5.7056	7.9987
1.4.10.tiff^45^	6.9216	7.9996

### Efficiency analysis

There are many factors that can affect the efficiency, such as the size of the image, the compression rate, and the degree of arithmetic power consumed by the encryption operation^57^. We select three images with sizes of 256$\times$256, 512$\times$512, and 1024$\times$1024, respectively, and encrypt and decrypt them respectively when the value of the quantization scale factor is $\alpha =2^{-3}$. Table 4 is obtained by measuring the time required for encryption, the time required for decryption and the image compression rate. As shown in Table 6, when the image size is larger, the required encryption and decryption time will increase accordingly. It can be seen from the experimental results that the overall encryption efficiency is acceptable.

**Table 6:** Encryption time comparison.

Picture	Encryption time (s)	Decryption time (s)	Compression ratio
5.1.11.tiff^45^(256256)	0.963761	0.558822	79.2969 %
motion01.512.tiff^45^(512512)	1.976872	0.846713	86.7188 %
$1^{\#}$(10241024)	15.394230	3.667921	82.4219 %

## Conclusion

This paper proposes an algorithm of high-quality restoration image encryption using Discrete Cosine Transform (DCT) frequency-domain compression coding and chaos. Firstly, the image hash value is used for the generation of an encryption key with plaintext correlation, then lightweight chaos is generated based on the key to obtain pseudo-random sequence. Secondly, partition the image into several $8\times 8$ subblocks, and perform DCT and quantization operations on all the subblocks respectively to obtain the DCT coefficient matrix. Next, extract the direct current (DC) coefficients and alternate current (AC) coefficients in the DCT coefficient matrix for compression coding to obtain two sets of bitstream containing DC coefficient and AC coefficient information. Then, permute the DC coefficient bit stream by the chaotic sequence, and perform ciphertext image reconstruction with the AC coefficient bitstream. Finally, the chaotic sequence is used to perform ciphertext diffusion, and the processed hash value is hidden in the ciphertext to obtain the final ciphertext. The theoretical and experimental analysis shows that the algorithm has the characteristics of high compression rate, high-quality image restoration large key space, strong plaintext sensitivity, strong key sensitivity and so on. Therefore, the method proposed in this paper can better improve the effectiveness and reliability of information in the transmission process, and is expected to provide a new idea for secure communication in the context of big data era.

## Data Availability

The datasets used and analysed during the current study available from the corresponding author on reasonable request.
